# P72. Transgenic expression of a chimeric signaling receptor to facilitate T cell costimulation in the tumour environment

**DOI:** 10.1186/2051-1426-2-S2-P46

**Published:** 2014-03-12

**Authors:** R Schlenker, M Leisegang, W Uckert, E Noessner

**Affiliations:** 1 Helmholtz Zentrum Muenchen, Institute of Molecular Immunology, Munich, Germany; 2 Max Delbrueck Center for Molecular Medicine, Molecular Cell Biology and Gene Therapy, Berlin, Germany

## 

Tumour therapy with T cell receptor (TCR) engineered T cells is reported to induce clinical responses but shortcomings regarding poor *in vivo* persistence and loss of function in the tumour milieu have been observed. Providing costimulation to adoptively transferred T cells may improve these shortcomings. However, human T effector cells are largely CD28 negative and epithelial tumours do not express CD80 or CD86. Therefore, costimulation of human CD8 T effector cells cannot be triggered via the classical way of CD28 ligation. We propose to facilitate costimulation of CD8 T effector cells in the tumour milieu through retroviral engineering of T cells with a chimeric signaling molecule (CSM). This CSM is consisted of an intracellular costimulatory domain fused to an extracellular domain with binding capacity for a ligand expressed by a great variety of tumours.

Human activated PBL retrovirally transduced to express the CSM exhibited a survival advantage during *in vitro* expansion according to clinical protocol. The effect of the chimeric molecule on T cell function was analyzed using T cells expressing a tumour antigen specific TCR alone or in combination with the CSM. Transduced T cells were stimulated with target cells positive or negative for the CSM ligand (CSM-L). CSM expressing T cells responded better to CSM-L^+^ target cells showing higher phosphorylation of ERK and RPS6 compared to stimulation with CSM-L^-^ target cells. CSM^-^ T cells responded equally to both target cells. Accordingly, CSM^+^ but not CSM^-^ T cells secreted more IL-2 and IFN-γ upon co-culture with CSM-L^+^ target cells. In summary, transduction of PBL with the chimeric signaling molecule supported T cell survival and TCR induced signaling leading to enhanced T cell function.

